# Effectiveness of cascade screening for elevated lipoprotein(a), an underdiagnosed family disorder

**DOI:** 10.1097/MOL.0000000000000951

**Published:** 2024-09-18

**Authors:** Maxim E. Annink, Emma S. Janssen, Laurens F. Reeskamp

**Affiliations:** Department of Vascular Medicine, Amsterdam Cardiovascular Sciences, Amsterdam University Medical Center, University of Amsterdam, Amsterdam, The Netherlands

**Keywords:** aortic valve stenosis, atherosclerotic cardiovascular disease, cardiovascular risk, familial cascade screening, lipoprotein(a)

## Abstract

**Purpose of review:**

Elevated lipoprotein(a) [Lp(a)] is a prevalent, independent, genetic risk factor for cardiovascular disease. Though crucial for adequate risk assessment, detection of individuals at increased risk because of elevated Lp(a) is severely lacking in practice. In this light, several consensus statements have recommended familial cascade screening strategies to increase detection of elevated Lp(a). This review aims to synthesize findings from recent research into the effectiveness of cascade screening for elevated Lp(a).

**Recent findings:**

Cascade screening is an effective method for identifying individuals with elevated Lp(a) and is superior to opportunistic screening. Cascade screening identifies approximately one new case of elevated Lp(a) ≥ 125 nmol/L for every two first-degree relatives screened. The number needed to screen (NNS) ranged from 1.3 to 2.9, depending on Lp(a) threshold values and selected population.

**Summary:**

Cascade screening appears to be a promising strategy for identifying individuals with elevated Lp(a). However, several challenges persist regarding the implementation of this strategy in clinical practice. Deciding on threshold values for initiating cascade screening, considering the implications of ethnicity-related variability of Lp(a) levels, and further research into the clinical relevance of cascade screening are crucial steps. Understanding these factors will be essential for optimizing cascade screening protocols and enhancing its effectiveness in clinical practice.

## Graphical abstract

**Figure:**
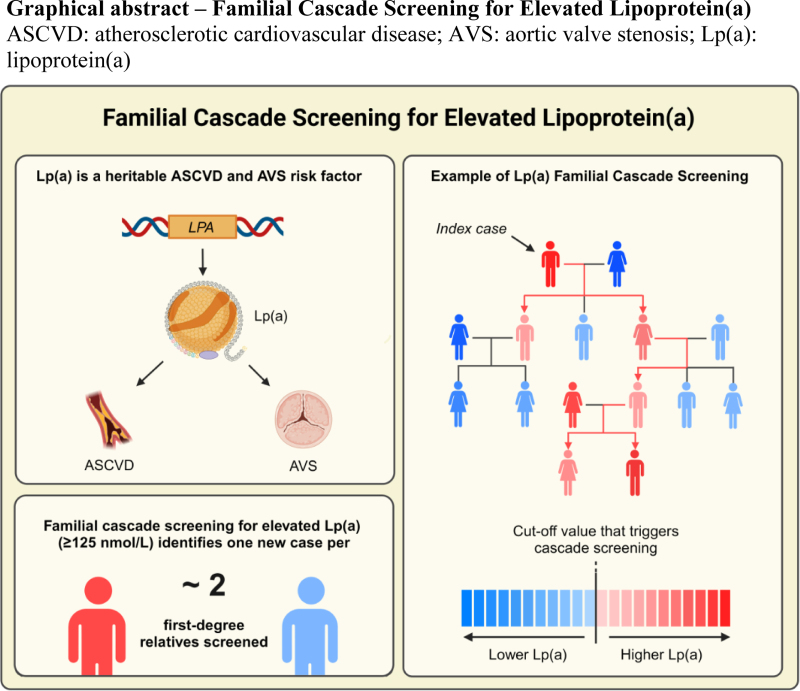

## INTRODUCTION

As atherosclerotic cardiovascular diseases (ASCVD) remain the leading cause of mortality worldwide [1], the challenge of accurately identifying at-risk individuals persists. This is illustrated by the fact that up to half of individuals in the general population have atherosclerosis in the absence of any classical cardiovascular risk factors [2]. Consequently, there is an urgent demand for the development of new, timely, and effective screening methods that enable physicians to identify individuals who may be predisposed to ASCVD in the future.

Over the six decades since its discovery, lipoprotein(a) [Lp(a)] has gained recognition as an independent and strong risk factor of ASCVD [3]. Lp(a) is a lipoprotein comprised of apolipoprotein B100 (apoB) with a linked apolipoprotein(a) [apo(a)] moiety [4]. Genetic variation at the *LPA* locus is the major determinant of Lp(a) levels. Overall, genetic variation, including multiple single nucleotide polymorphisms (SNPs) and, most importantly, the number of Kringle IV repeats, is responsible for 70–90% of the interindividual variance in Lp(a) concentrations [5–8]. As a result, Lp(a) concentrations remain relatively stable throughout adulthood and are not influenced by lifestyle factors [9].

Previously, Mendelian randomization studies have established that Lp(a) is most likely causally linked to the development of ASCVD [10,11]. Unlike low-density lipoprotein (LDL), whose primary contribution to atherogenesis is governed by accumulation of particles and their cholesterol content in the vascular wall, the pathogenicity of Lp(a) is mediated by its pro-atherogenic and pro-inflammatory cell signaling effects [12]. Of note, Lp(a) has a large binding capacity for oxidized phospholipids (OxPLs), which induce an inflammatory state in the vascular wall and promote monocyte migration into the endothelium, driving atherogenesis [13–15]. In addition to being a risk factor for ASCVD, there is growing evidence that Lp(a) is positively associated with the presence of aortic valve calcification, as well as progression to aortic valve stenosis (AVS) [16,17]. Consequently, elevated Lp(a) levels increase the risk of requiring aortic valve replacement and mortality [18,19].

The epidemiological importance of detecting individuals with elevated Lp(a) levels is underscored by the fact that, depending on ethnicity, approximately one-fifth of the population exhibit elevated Lp(a) levels, defined in international consensus statements as at least 125 nmol/l with an associated 1.25-fold increased lifetime risk of ASCVD [20,21]. Beyond this dichotomous risk classification, Lp(a) concentrations are linearly associated with ASCVD risk [20]. It is unsurprising, therefore, that measurement of Lp(a) at least once per lifetime is now recommended by several scientific organizations [20–22]. Finding elevated Lp(a) will potentially become all the more important in years to come, as phase 2 trials of Lp(a)-lowering agents have yielded impressive reductions of Lp(a) levels of up to 92–98% [23], with large phase III cardiovascular outcome trials currently underway.

Yet, it is clear that the implementation of strategies to identify individuals with elevated Lp(a) that could benefit from these therapies is lagging behind. For instance, 14% of patients with known ASCVD had a prior measurement of Lp(a) in a large international cohort study [24]. In some regions, routine Lp(a) testing is virtually nonexistent. Examples include Germany (<1% of patients with known ASCVD) and Israel (0.1% of patients within a health maintenance organization) [25,26]. An efficient way to increase the uptake of routine Lp(a) testing could be to leverage the heritability of Lp(a) by employing a familial cascade screening strategy, inspired by the successful genetic cascade testing programs that are in place for familial hypercholesterolemia [27]. Within cascade screening programs, first-degree relatives (i.e. parents, siblings, and children) of individuals with a given trait [e.g. familial hypercholesterolemia or elevated Lp(a)] are tested for the presence of this trait as well. An iterative process is then initiated where first-degree relatives of the individuals that were identified in the previous screening round, are also investigated for carriership of the given trait (Graphical abstract). This review aims to provide an overview of the current knowledge on cascade screening for elevated Lp(a). Multiple studies that have investigated the effectiveness of cascade screening are examined, and various trade-offs associated with its implementation are explored. 

**Box 1:**
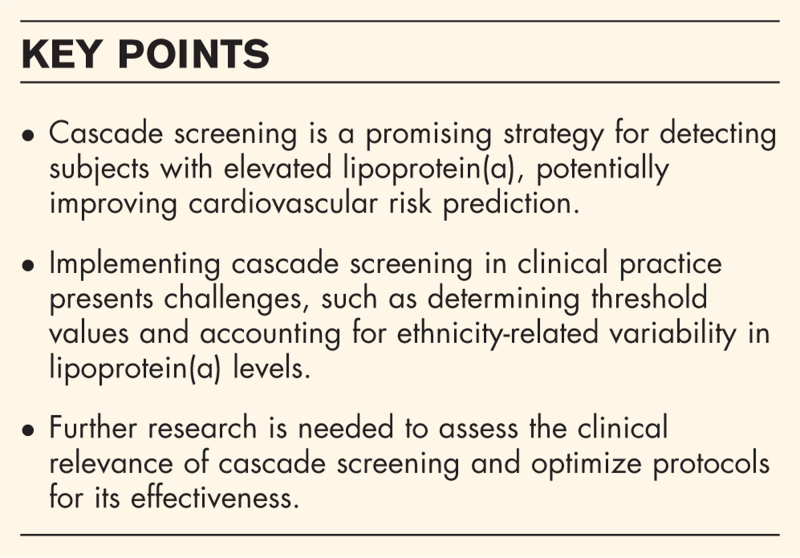
no caption available

## LIPOPROTEIN(a) CASCADE SCREENING

In recent years, an increasing number of studies have assessed the yield of cascade screening programs in different study populations, using varying cutoff levels. As this is explicitly recommended in current guidelines, many of these studies have made opportunistic use of already existing and widely implemented genetic cascade screening programs for familial hypercholesterolemia to screen for elevated Lp(a) concomitantly. For instance, Loh *et al.*[28^▪▪^] examined the yield of measuring Lp(a) in a small Australian cohort of 103 children and siblings aged 18 years or younger of 66 adult index cases with genetically confirmed familial hypercholesterolemia. The median age of the relatives was 12 years. A cutoff value of at least 50 mg/dl (∼125 nmol/l) was used to select index cases, resulting in the detection of one new case of elevated Lp(a) (≥30 mg/dl; ∼75 nmol/l) for every two individuals screened (Table 1). Conversely, the yield of testing relatives of index cases with normal Lp(a) (i.e. ≤50 mg/dl; ∼125 nmol/l) was significantly lower, at one person with elevated Lp(a) (≥30 mg/dl; ∼75 nmol/l) for every 7.5 individuals screened. Of particular interest was the fact that no less than four out of five children Lp(a) levels at least 100 mg/dl (∼250 nmol/l) had elevated Lp(a) levels themselves. Despite differences in cutoffs for selection of index patients and defining elevated Lp(a) in first-degree relatives (50 mg/dl; ∼125 vs. 30 mg/dl; ∼75 nmol/l), this study nicely demonstrated that elevated Lp(a) levels can already be detected through cascade screening in childhood.

**Table 1:** Summary of reviewed studies into lipoprotein(a) cascade screening

Reference	Setting	Lp(a) index	*n* indices	Lp(a) relative	Degree of relatedness	Relatives with elevated Lp(a) (%)	NNS
Loh *et al.*[28^▪▪^]	Cohort of adult FH patients and their children and sibling aged ≤18 years	<50 mg/dl	50	≥30 mg/dl	First degree	13.3%^a^	7.5
		30–49 mg/dl	9			27.8%	3.6^a^
		≥50 mg/dl	16			50.0%	2.0
		≥100 mg/dl	3			80.0%	1.3^a^
Ellis *et al.*[30]	Longitudinal cohort of FH patients and their relatives	<50 mg/dl	533	≥50 mg/dl	First degree	17.0%	5.9
					Any degree	17.1%	5.8
		≥50 mg/dl	222		First degree	50.1%	2.0
					Any degree	42.1%	2.4
Chakraborty *et al.*[31]	Cohort of adult FH patients and their relatives	≥50 mg/dl	62	≥50 mg/dl	First degree	49.1%	2.0^a^
		≥50 mg/dl	62	≥50 mg/dl	Any degree	41.4%	2.4^a^
		≥50 mg/dl	62	≥100 mg/dl		34.3%	2.9^a^
		50–99 mg/dl	n.r.	≥50 mg/dl		34.0%	2.9^a^
		≥100 mg/dl	n.r.	≥50 mg/dl		53.0%	1.9
Chakraborty *et al.*[32]	Cohort of adult patients with phenotypic dyslipidemias and elevated Lp(a) and their relatives	≥100 mg/dl	83	≥50 mg/dl	Any degree (94% first degree and 6% second degree)	68.1%	1.5
				≥100 mg/dl		35.2%	2.8
		100–150 mg/dl	41	≥50 mg/dl		55.5%	1.8
				≥100 mg/dl		27.2%	3.7^a^
		≥150 mg/dl	42	≥50 mg/dl		81.1%	1.2
				≥100 mg/dl		43.3%	2.3^a^
Reeskamp *et al.*[33^▪▪^]	Participants of the UK biobank, with relatedness construed from genetic relationships	≥125 nmol/l	3420	≥125 nmol/l	First degree	47.0%	2.1
					Second degree	31.8%	3.1
		≥150 nmol/l	1609	≥150 nmol/l	First degree	41.6%	2.4^a^
					Second degree	27.7%	3.6^a^
		≥200 nmol/l	1333	≥200 nmol/l	First degree	34.3%	2.9^a^
					Second degree	19.4%	5.2^a^

FH, familial hypercholesterolemia; Lp(a), lipoprotein(a); NNS, number needed to screen; n.r., not reported.

a Value was not reported in publication, but was calculated from available data.

Comparable findings were observed in a study that selected index patients from the longitudinal Spanish Familial Hypercholesterolemia Cohort Study (SAFEHEART) [29]. Ellis *et al.*[30] recruited 755 index patients and screened 2927 relatives irrespective of degree of relatedness with a median age of 43.6 years old, of which 8.8% had a history of ASCVD. Here, too, a threshold value of at least 50 mg/dl (∼125 nmol/l) was used to identify index cases, which resulted in a yield of one new case of elevated Lp(a) (≥50 mg/dl; ∼125 nmol/l) for every 2.4 relatives screened. Limiting the cascade screening strategy to first-degree relatives improved the yield to one in two relatives. Unsurprisingly, the yield of screening in index familial hypercholesterolemia patients without elevated Lp(a) was much lower, with one new case (≥50 mg/dl; ∼125 nmol/l) identified for every 5.8 relatives screened, corresponding roughly to the prevalence in the general population. Speaking to the clinical significance of Lp(a) cascade screening, relatives with elevated Lp(a) and no familial hypercholesterolemia were at significantly elevated risk of reaching a composite endpoint of ASCVD (defined as any coronary, cerebrovascular, or peripheral arterial obstructive disease or revascularization) and cardiovascular mortality compared with relatives with normal Lp(a) and no familial hypercholesterolemia during a median follow-up time of 4.4 years after screening (hazard ratio: 3.17; 95% CI: 1.16–8.64; *P* = 0.024). This increased risk was similar in magnitude to that of relatives with familial hypercholesterolemia alone (hazard ratio: 2.47; 95% CI 1.06–5.74; *P* = 0.036).

Chakraborty *et al.* carried out Lp(a) cascade screening within an Australian genetic testing program for familial hypercholesterolemia, where once more, a threshold of at least 50 mg/dl (∼125 nmol/l) was used for both index cases and relatives [31]. They recruited 62 index patients and 162 relatives of any degree of relatedness and age, including 26 minors. 22.8% of adult relatives had a history of ASCVD. This strategy yielded approximately one new case for every 2.4 relatives screened. Limiting screening to first-degree relatives led to a higher detection rate of approximately one new case for every two relatives tested. Additionally, a higher detection rate was achieved when a threshold of Lp(a) of at least 100 mg/dl (∼250 nmol/l) was applied to the index patients, yielding approximately 1 out of 1.9 relatives tested with elevated Lp(a) of at least 50 mg/dl (∼125 nmol/l) compared with 1 out of 2.9 when the index threshold was set between 50 and 99 mg/dl (∼125 and ∼247.5 nmol/l, respectively).

Only two studies have investigated the effectiveness of Lp(a) cascade screening, independently of familial hypercholesterolemia screening. Chakraborty *et al.* conducted another study in a cohort of 83 probands with phenotypic dyslipidemias, excluding familial hypercholesterolemia, treated at an Australian tertiary lipid clinic. One hundred and eighty-two relatives were included, including 31 children and adolescent relatives [32]. All probands had plasma Lp(a) concentrations of at least 100 mg/dl (∼250 nmol/l), and their relatives were tested for elevated Lp(a) (defined as ≥50 mg/dl ∼125 nmol/l). The average age of the adult relatives was 41.1 years old, with 8% having a history of CAD (defined as myocardial infarction, coronary angioplasty or stenting, coronary artery bypass grafting or angina pectoris). The average age of children and adolescent relatives was 12.7 years old, with none having a history of CAD. Elevated Lp(a) concentrations (≥50 mg/dl and ≥100 mg/dl; ∼125 nmol/l, ∼250 nmol/l) were detected in one new case for every 1.5 and 2.8 relatives tested, respectively. At higher Lp(a) cutoff values for indices of 100–150 mg/dl and ≥150 mg/dl (∼250 to ∼375 nmol/l, ≥375 nmol/l), respectively, 1 out of 1.8 and 1 out of 1.2 relatives had elevated Lp(a) of at least 50 mg/dl (∼125 nmol/l).

**FIGURE 1:**
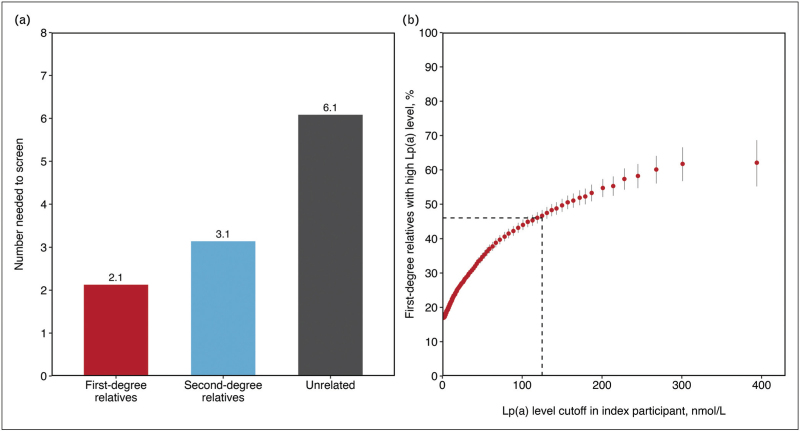
Concordance of elevated lipoprotein(a) levels and number needed to screen among relatives of index participants with elevated lipoprotein(a) levels in the UK Biobank. The estimated number needed to screen for elevated Lp(a) (≥125 nmol/l) in the UK Biobank for first-degree relatives was 2.1 and second-degree relatives 3.1. In contrast, the number needed to screen for unrelated individuals was much higher 6.1 (panel a). With increasing Lp(a) cutoff levels in the index patients, the percentage of first-degree relatives with elevated Lp(a) in the UK Biobank gradually increases (panel b). Reproduced with permission from Reeskamp *et al.*[33^▪▪^]. Copyright © (2023) American Medical Association. All rights reserved, including those for text and datamining, AI training, and similar technologies.

Finally, Reeskamp *et al.*[33^▪▪^] investigated the potential yield of cascade screening for elevated Lp(a) in first-degree and second-degree relatives in the general population, by leveraging the genetic information on relatedness and biochemical data of the UK Biobank participants. This cross-sectional study analyzed the concordance of Lp(a) levels of 19 899 first-degree relative pairs, 9715 second-degree relative pairs, and 184 764 unrelated pairs as a control group. The first-degree and second-degree relatives had a mean age of 57.3 years, with 4.6% of first-degree and 5.6% of second-degree relatives having a history of ASCVD. The control group exhibited similar characteristics, with a median age of 57.1 years and 4.9% having a prior history of ASCVD. A threshold level of at least 125 nmol/l (∼50 mg/dl) was utilized for index participants, resulting in a hypothetical yield of one new case (≥125 nmol/l) for every 2.1 first-degree relatives tested and for every 3.1 second-degree relatives tested (Fig. 1a). The continuous and nondichotomous nature of concordance of elevated Lp(a) among relatives is illustrated by the observation that the percentage of first-degree relatives with high Lp(a) (>125 nmol/l) continuously increases with higher Lp(a) concentrations in the proband (Fig. 1b). The concordance with a threshold for elevated Lp(a) for relatives of at least 200 nmol/l (∼80 mg/dl) resulted in a yield of one new case for every 2.9 first-degree relatives tested and 5.2 second-degree relatives tested. In comparison, the yield among unrelated individuals was one new case for every 6.1 individuals tested. Importantly, the detection rate of elevated Lp(a) was similar among male and female individuals, those using statins or other lipid-lowering therapies, women on hormone replacement therapy, or postmenopausal women. The detection rate was generally higher (although not statistically significant) in relatives of Black and South Asian ethnicities compared with whites and in those who were carrier of two risk alleles for high Lp(a) (rs1045872 and rs3798220).

## DISCUSSION

In light of the growing consensus that Lp(a) represents a significant and actionable risk factor for ASCVD and AVS [34], current guidelines advocate universal screening strategies by urging healthcare professionals to measure Lp(a) in every adult at least once per lifetime [20,21]. In order to accelerate the identification of those who are at heightened risk of ASCVD, an increasing number of consensus statements recommend the use of familial cascade screening strategies to identify those with elevated Lp(a) [20,21,35]. This review aimed to synthesize the current evidence supporting this recommendation. In summary, the number of first-degree relatives needed to screen in these studies ranged from 1.3 to 2.9, depending on the selected population and Lp(a) threshold values (Table 1). These findings support the use of Lp(a) familial cascade screening as an effective measure to identify those with elevated Lp(a). However, several challenges remain regarding the implementation of this strategy in clinical practice.

First, mention should be made of the fact that results from phase III trials investigating the effects on ASCVD risk of specific Lp(a)-lowering agents are still pending. If positive, these trials will definitively prove that Lp(a) is an actionable and causal target for ASCVD risk reduction. Awaiting the results from these trials, however, early and intensive modification of other ASCVD risk factors such as obesity, smoking, LDL cholesterol, and hypertension in those with elevated Lp(a) is widely recommended [20,21]. This recommendation is based on the observation that elevated Lp(a) levels amplify the risk conferred by these risk factors [36]. It has even been suggested that Lp(a)-associated ASCVD risk can be attenuated by lowering LDL cholesterol by an amount proportional to the magnitude of that risk [20]. In order to achieve clinical implementation of such strategies, it is, therefore, imperative to carefully design care pathways for individuals with elevated Lp(a) who are identified through cascade screening programs. These pathways should include a comprehensive cardiovascular risk assessment and the initiation of appropriate treatment, especially as the majority of these individuals will likely be asymptomatic and, therefore, lack a previously recorded cardiovascular risk profile. Furthermore, Lp(a) cascade screening programs will require careful risk communication to those who consider being screened and especially to those who are subsequently identified as having elevated Lp(a). This is an area that requires further study in the context of Lp(a) cascade screening.

Second, given that the risk imparted by Lp(a) concentrations is linear, providing a singular and meaningful definition of elevated Lp(a) in clinical practice is complicated. In this regard, the EAS and NLA have pragmatically recommended a threshold of at least 125 nmol/l (∼ ≥50 mg/dl) to indicate elevated ASCVD risk [20,21]. As this threshold corresponds to approximately one-fifth of the population, the achievability of familial cascade screening maintaining a similar cutoff value in index patients is questionable. Several studies under review here have demonstrated that the number of relatives needed to screen to find elevated Lp(a) is more favorable when a higher threshold value is selected for indices. This outcome is not unexpected, given that higher Lp(a) levels in indices correlate with higher Lp(a) levels in their relatives. Furthermore, from a resource allocation perspective, increasing the Lp(a) thresholds for initiating cascade screening improves the feasibility of this strategy by reducing the number of individuals in which cascade screening is initiated, while still identifying those with the highest risk. It appears reasonable, therefore, to suggest that clinicians implementing this strategy should prioritize cascade screening in those with the highest of Lp(a) levels. Remarkably, a recent article showed that risk conferred by high Lp(a) levels might be different in patients with and without prior ASCVD. In secondary prevention settings, this risk appears to only marginally increase at levels of Lp(a) above 150 nmol/l (∼60 mg/dl), implying that cascade screening programs might be most meaningful for identification of individuals with elevated Lp(a) but without prior ASCVD [37].

Third, epidemiological studies have consistently demonstrated that Lp(a) levels are ethnicity dependent with higher median concentrations observed in Black and South Asian individuals compared to White individuals [38–40]. These variations should be considered during the implementation of cascade strategies to avoid misclassification and ensure equitable care. As Reeskamp *et al.* demonstrated, ethnic differences in Lp(a) concentration potentially affect the detection rate of elevated Lp(a) in relatives, resulting in a lower number needed to screen in Black and South Asian individuals. This remains a topic of future research, however, as none of the other studies have included ethnicity-specific data because of limited population sizes. It is important to note that despite the differences in baseline Lp(a) levels, the risk of ASCVD associated with Lp(a) concentration was found to be broadly similar across ethnic groups in the UK Biobank study [38]. Although applying race-specific percentiles in this study provided more consistent risk estimates across racial groups, the absolute differences between these threshold approaches were minor. Given the clinical practicality of a uniform cutoff value, this finding might warrant the use of a uniform cutoff value for initiating cascade screening across different ethnic groups.

Finally, future research efforts should be directed towards the assessment of the cardiovascular status of relatives identified with elevated Lp(a). Ellis *et al.*[30] have demonstrated that relatives with elevated Lp(a) are at elevated risk for cardiovascular disease, emphasizing the clinical significance of cascade screening. Nevertheless, additional research is required to substantiate these findings and to ascertain the extent to which elevated Lp(a) levels in relatives are associated with an increased risk of ASCVD. Future research could therefore focus on evaluating the cardiovascular event rates or markers of subclinical atherosclerosis, such as coronary artery calcium scoring (CACS) or CCTA imaging, in relatives with elevated Lp(a). To most effectively assess the value of this approach in the general population, these investigations should be conducted in relatives of individuals with isolated Lp(a) elevation, separately from existing familial hypercholesterolemia cascade screening programs.

## CONCLUSION

Cascade screening for elevated Lp(a) is likely effective in both familial hypercholesterolemia patients and in the general population. Whether this identifies patient at increased risk or with subclinical atherosclerosis needs to be established, especially as no specific Lp(a) therapies are available. Regardless of age and ethnicity, Lp(a) cascade screening appears to be feasible and should be offered in clinical practice. Nationwide screening programs might facilitate large-scale detection of high Lp(a) similar to the success of nationwide cascade screening programs for familial hypercholesterolemia.

## Acknowledgements

*None.*

### Financial support and sponsorship

*This work was supported by a research grant from Klinkerpadfonds.*

### Conflicts of interest

*L.F.R. is co-founder of Lipid Tools and has received speaker fees from Ultragenyx, Daiichi Sankyo, and Novartis.*

## REFERENCES AND RECOMMENDED READING

Papers of particular interest, published within the annual period of review, have been highlighted as:

▪ of special interest
▪▪ of outstanding interest
